# Genes predict long distance migration and large body size in a migratory fish, Pacific lamprey

**DOI:** 10.1111/eva.12203

**Published:** 2014-09-23

**Authors:** Jon E Hess, Christopher C Caudill, Matthew L Keefer, Brian J McIlraith, Mary L Moser, Shawn R Narum

**Affiliations:** 1 Columbia River Inter-Tribal Fish CommissionHagerman, ID, USA; 2 Department of Fish and Wildlife Sciences, College of Natural Resources, University of IdahoMoscow, ID, USA; 3 Columbia River Inter-Tribal Fish CommissionPortland, OR, USA; 4 Fish Ecology Division, Northwest Fisheries Science Center, National Marine Fisheries Service, National Oceanic and Atmospheric AdministrationSeattle, WA, USA

**Keywords:** anadromous fishes, association study, hydrosystem, migratory species, translocation

## Abstract

Elucidation of genetic mechanisms underpinning migratory behavior could help predict how changes in genetic diversity may affect future spatiotemporal distribution of a migratory species. This ability would benefit conservation of one such declining species, anadromous Pacific lamprey (*Entosphenus tridentatus*). Nonphilopatric migration of adult Pacific lamprey has homogenized population-level neutral variation but has maintained adaptive variation that differentiates groups based on geography, run-timing and adult body form. To investigate causes for this adaptive divergence, we examined 647 adult lamprey sampled at a fixed location on the Columbia River and radiotracked during their subsequent upstream migration. We tested whether genetic variation [94 neutral and adaptive single nucleotide polymorphisms (SNPs) previously identified from a genomewide association study] was associated with phenotypes of migration distance, migration timing, or morphology. Three adaptive markers were strongly associated with morphology, and one marker also correlated with upstream migration distance and timing. Genes physically linked with these markers plausibly influence differences in body size, which is also consistently associated with migration distance in Pacific lamprey. Pacific lamprey conservation implications include the potential to predict an individual's upstream destination based on its genotype. More broadly, the results suggest a genetic basis for intrapopulation variation in migration distance in migratory species.

## Introduction

Genetic mechanisms underpinning various aspects of migratory behavior have been discovered in animals that traverse great distances and display precise homing ability (e.g., timing, sun compass orientation, and propensity to migrate; Zhu et al. 2009; Hecht et al. 2013; O'Malley et al. 2013). Characterization of particular genetic traits may increase our ability to predict the spatial and temporal distribution of migratory species and could thus increase effectiveness of the management of such species, many of which play key cultural and economic roles in our society. Effective management is particularly challenging for migratory species that have unpredictable movement (e.g., do not home to their natal site).

Genetic tools to help predict various aspects of migration could benefit conservation of one such species, anadromous Pacific lamprey (*Entosphenus tridentatus*). Recent genetic surveys indicate that the species has seemingly nonphilopatric migration of adults from ocean feeding sites to freshwater spawning sites based on homogenized neutral variation across broad geographical regions (Spice et al. 2012; Hess et al. 2013). Yet this species maintains adaptive variation that differentiates groups based on geography, run-timing, and adult body form (Hess et al. 2013). Severe declines in abundance of Pacific lamprey have occurred throughout its range in the Pacific Northwest of the USA, including the Columbia River Basin (Close et al. 2002), where multiple anthropogenic factors (e.g., artificial barriers and past extermination practices) likely contributed to the species' extirpation in various tributaries of the interior (Close et al. 2009). These declines have prompted trap and haul translocation efforts as a conservation strategy to help recover Pacific lamprey abundance and restore its key role in the ecosystem while providing sustainable harvest in the interior Columbia River (Close et al. 2009; Ward et al. 2012). The minimal neutral population structure may be a factor that aids widespread adoption of a translocation-type strategy (i.e., transporting individuals from a source population to a recipient site) because it precludes need for consideration of one potential risk of this strategy (i.e., disruption of population structure; Weeks et al. 2011). Yet, what would be the management implications of pronounced adaptive divergence in this basin? What fitness effects (if any) would occur if translocations were to alter the frequencies of particular adaptive genetic variants in different regions of the basin? How might adaptive gradients influence the effectiveness of translocations? Some of these adaptive loci have been observed to display dramatic shifts in minor allele frequencies (MAFs) between the lower Columbia River and interior Columbia River (Hess et al. 2014a), which represent the source and recipient regions for translocation, respectively. Therefore, the probability for altering adaptive allele frequencies via Pacific lamprey translocation is high, creating added incentive to gain an understanding of the mechanisms driving this adaptive divergence and how it relates to the biology of this migratory species.

Hess et al. (2013) identified 162 of 4439 quality-filtered restriction site–associated DNA sequencing (RAD) single nucleotide polymorphisms (SNPs) as *F*_ST_ outliers. Further, significant linkage among pairs of loci made it possible to categorize the adaptive loci into four groups of linked loci, and a subset of four SNPs were selected such that each SNP represented one of the four groups of linked loci (Hess et al. 2013). These four SNPs and five other adaptive SNPs were incorporated into a marker panel optimized for multiple conservation applications, including characterization of adaptive variation (Hess et al. 2014a). Hess et al. (2014a) showed that body lengths of Pacific lamprey captured at Willamette Falls (Willamette River, OR, USA) significantly correlated with the MAF of two adaptive loci (*Etr_5317*, *Etr_1806*), and run-timing of these lamprey is also significantly correlated with one of these same markers (*Etr_1806*). Further, these two loci displayed a steep gradient in MAF between the lower Columbia River and interior Columbia River. However, observed correlations between these two loci and phenotypes of lamprey at Willamette Falls may be confounded by life-history diversity unique to that tributary. Specifically, that early-migrating, short-bodied lamprey may be primarily composed by lamprey that have overwintered in the river, as opposed to those that have recently entered freshwater (Clemens et al. 2010; Hess et al. 2014a). Therefore, it is not clear whether association with length or passage timing explained the genetic divergence observed on a larger scale (Columbia River Basin). A more comprehensive examination of these potential correlations was warranted.

Bonneville Dam is the first dam anadromous fish encounter as they migrate from the Pacific Ocean to the interior of the Columbia River (river km 235), and it provides an appealing site at which to collect, measure variable phenotypic and genetic traits, and track the movement of Pacific lamprey through the interior Columbia River. This site houses an adult fish facility where Pacific salmonids (*Oncorhynchus* spp) and Pacific lamprey are routinely sampled to address dam passage questions (e.g., Moser et al. 2002a,b; Johnson et al. 2012) and to perform genetic monitoring of stock-specific abundance and run-timing (e.g., Hess et al. 2014b). Further, this location coincides with a steep genetic transition observed for many adaptive loci in Pacific lamprey (Hess et al. 2013, 2014a). Finally, past work by Keefer et al. (2009, 2013a) has noted an important interaction between the size of lamprey and migration behavior, in that relatively large-bodied Pacific lamprey typically arrive earlier at Bonneville Dam and travel further upstream than small-bodied lamprey in the same year. Unlike the Willamette River, an interaction between size and migration timing is less likely to be confounded by effects of overwintering behavior prior to collection, which is rare (<5%) among adults collected at Bonneville Dam.

The primary objective of this study was to test for SNPs associated with migration distance, migration timing, and morphology using individual-based data gathered from Pacific lamprey captured at Bonneville Dam. Any locus identified as being significantly associated with one or more of these classes of predictor variables was then included in a multivariate analysis to determine the combination of predictor variables that best explained observed genetic variation. We also pursued a secondary objective as a way to relate our primary findings to the translocation strategy being used in conservation efforts for this species. Our secondary objective was to determine whether a set of Pacific lamprey collected at Bonneville Dam for translocation were distinguished morphologically and genetically from Pacific lamprey either selected randomly at different points in the migration season, or from Pacific lamprey known to have volitionally migrated to monitoring stations near the translocation site.

## Methods

### Pacific lamprey collection, tagging, and monitoring

**Figure 1:**
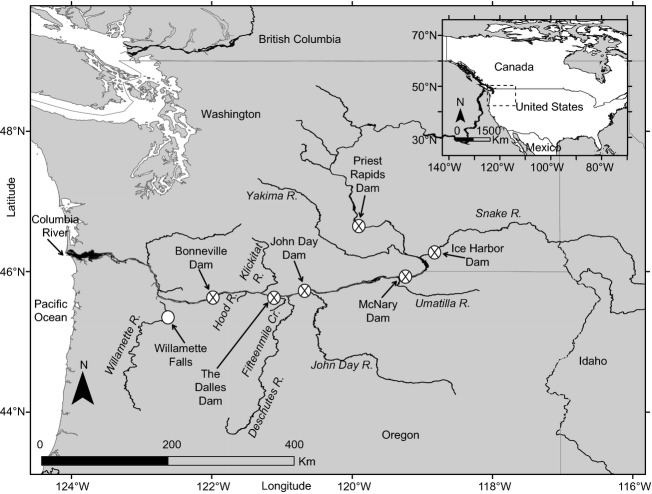
Map of study area. The fate categories included below Bonneville Dam, Bonneville Dam to John Day Dam (within main stem or secondary tributary), above John Day Dam, and above Ice Harbor Dam (Snake River). The Umatilla River is the tributary where the collection of individuals used for translocation were released to supplement the interior Columbia River population. Collection/detection sites at dams and nondams are shown with X's and open circles, respectively.

Adult Pacific lamprey were collected inside fishways at Bonneville Dam on the Columbia River (46°N, 124°W; Fig. 1) using passive traps that were deployed at night from early June through early September, 2008–2010. Details of trapping, anesthesia, and surgical methods were described by Moser et al. (2002a,b) and Johnson et al. (2012). All fish were anesthetized (60-ppm eugenol solution) measured (total length and girth at first dorsal insertion to the nearest mm), weighed (nearest g), and scanned with a Distell Fatmeter (Distell Industries, Ltd., Lothian, Scotland; Crossin and Hinch 2005). Lamprey with girth >9 cm were radiotagged in approximate proportion to the run in each year and sampled fish were randomly selected from those that were trapped insomuch as possible. In 2008, a portion of the untagged Pacific lamprey of all sizes was used for translocation to the Umatilla River (Close et al. 2009). This study also utilized data from Hess et al. (2014a) who captured 150 adult Pacific lamprey at the entrance to the fish ladder at Willamette Falls (Fig. 1): a random sample of 50 Pacific lamprey taken from three time periods (2-week intervals) at peaks in the multi-modal abundance distribution observed in 2012 (April–August). Hess et al. (2014a) recorded passage timing in units of statistical week of collection and measured each individual for total body length (mm).

For Bonneville Dam tagging, a uniquely coded radio transmitter (Model NTC-4-2L, 8 × 18 mm, 2.1 g; Lotek Wireless, Newmarket, ON, Canada) was placed inside the body cavity with its trailing antenna threaded through the body wall using a cannula or catheter. A secondary tag, a 4 × 32 mm half-duplex passive integrated transponder (PIT), was inserted into the body cavity of each fish to assess transmitter loss and failure. After tagging, Pacific lamprey recovered in a flow-through tank supplied with river water for at least 2 h. They were transported by truck to release sites located about 3 km downstream from Bonneville Dam, one on each bank. Pacific lamprey were released in approximately equal proportions and release site assignment was random with respect to individual fish (release sites alternated between tag groups). All handling and tagging protocols were reviewed and approved by the University of Idaho Animal Care and Use Committee and conducted under state scientific collection and transportation permits.

Tagged Pacific lamprey was monitored using an extensive array of aerial and underwater radio antennas (e.g., Keefer et al. 2013a,b). Underwater antennas were deployed outside fishway openings, inside fishways, and at fish ladder exits at Bonneville, The Dalles, John Day, and McNary dams on the lower Columbia River, Priest Rapids Dam on the upper Columbia River, and Ice Harbor Dam on the lower Snake River. Underwater antennas had detection ranges up to approximately 10 m. Aerial radio antennas with longer detection ranges (up to ∼1 km) were deployed in dam tailraces and at the mouths of 14 major Columbia River tributaries between the Willamette River confluence and Priest Rapids Dam (Fig. 1): Willamette River, Eagle Creek, Herman Creek, Fifteenmile Creek, Rock Creek, Wind River, Little White Salmon River, White Salmon River, Hood River, Klickitat River, Deschutes River, John Day River, Yakima River, and Umatilla River. Additionally, PIT tag detection antennas monitored lamprey passage at the main stem dams (Keefer et al. 2009) and at in-stream sites in the Hood River, Fifteenmile Creek, and the Deschutes River basin.

A detection history for each tagged lamprey was generated using an automated program that assigned activity codes to time-stamped detections at each radio antenna. Potential ‘noise’ records were excluded using filters that identified signal collisions. Each history was then reviewed by an experienced technician who further identified records that did not have corroborating support from detections at nearby sites. A final ‘fate’ was assigned to each lamprey using the last plausible radiotelemetry or PIT tag detection record. A majority of final detections were at Columbia River dams or reservoirs. This was an artifact, at least in part, of radio transmitter battery life. Most Pacific lamprey overwinter in freshwater (including reservoirs) and then move to tributary spawning sites in the spring (Robinson and Bayer 2005; Clemens et al. 2010), and the elapsed time between tagging and springtime tributary entry typically exceeded the 162-d battery life (5 s burst rate). Thus, we note that the sampled population may have been biased because not all lampreys could be tagged due to size restrictions and other logistical constraints and because we may have underestimated migration distance in some adults. However, both potential biases (e.g., fate misclassification) should act to decrease any observed effects of body size or fate (i.e., increase the probability of a Type II error) rather than create false differences.

### Genetic data collection

Tissues were collected from the dorsal fin of each adult Pacific lamprey and stored in a buffered solution before DNA extractions were performed with Qiagen DNeasy kits (Qiagen, Valencia, CA, USA). We used a set of 96 SNP loci to genotype 647 individual adult lamprey that were sampled at Bonneville Dam and radio-tracked during their upstream migration. This set of 96 SNPs (94 of which are highly polymorphic in Pacific lamprey) was developed following a genomewide association study (Hess et al. 2013), which used *F*_ST_ outlier tests to determine that 85 and 9 of the SNPs were putatively neutral and adaptive, respectively. For the genomewide association study, a RAD catalog was constructed using a subset of individuals (6–12 total individuals from 3 to 6 collections) and then used to align and perform genotyping by sequencing on a larger sample of individuals (*N* = 518) from 21 collections distributed throughout the Pacific Northwest range of Pacific lamprey (Hess et al. 2013). Subsequently, Hess et al. (2014a) demonstrated that this set of 96 SNPs could perform the following three critical applications in Pacific lamprey: (i) species identification, (ii) parentage assignment, and (iii) characterization of adaptive and neutral variation. These 96 SNPs were genotyped using TaqMan assays supplied by Applied Biosystems (Grand Island, NY, USA). Genotypic data were collected using the Fluidigm EP-1 96.96 system and following the manufacturer's suggested protocol, but modified slightly by including a sample preamplification step and increasing the PCR cycles to 50 (Hess et al. 2014a). All 647 individuals included in this study passed quality-filtering using a missing data threshold of 10% (i.e., <10 missing SNPs/individual).

### Statistical analyses

Linkage disequilibrium (LD) and Hardy–Weinberg equilibrium (HWE) deviations based on *F*_IS_ were assessed using a subset of the dataset (515 Pacific lamprey), split among 3 years 2008–2010. The Markov Chain Monte Carlo approximation of Fisher's exact test was used to test LD for all pairs of loci (94 SNPs) within each year sample using GENEPOP v. 4.1 (Raymond and Rousset 1995), with default parameter settings of 10 000 dememorizations, 100 batches, and 5000 iterations. FSTAT v. 2.9.3.2 (Goudet 2002) was used to calculate *F*_IS_ for each year sample, and 282 000 randomizations were used to test significance of each value. A Bonferroni correction was applied to significance levels (1.77 × 10^−4^, initial *α* = 0.05) for both *F*_IS_ and tests of LD to reduce false positives from multiple tests.

This mixed group of fish was used to test HWE deviations because previous studies have not identified more than a single randomly mating population above Bonneville Dam using neutral markers (Spice et al. 2012; Hess et al. 2013). These Pacific lamprey were randomly selected at an approximately 2% average sample rate across the majority of the run of Pacific lamprey observed at Bonneville Dam during 2008–2010 (Table 1). Most (98%) of the 515 Pacific lamprey had final location fates (Table 2); however, not all fate categories were adequately represented in this random sample, and so we added 62 Pacific lamprey that were nonrandomly selected to obtain a minimum of 50 Pacific lamprey in each fate category (Table 2). This total ‘Bonneville’ sample (*N* = 577) was used to test whether genetic variation was associated with migration fate, migration-timing, or morphology predictor variables; however, we also analyzed each of the 3 year samples separately to test for the consistency of associations across years. Finally, an additional set of 70 Pacific lamprey was also genotyped and was used to address our secondary objective (the ‘translocation’ sample). These 70 Pacific lamprey were used in translocations in 2008 to supplement the interior portion of the Columbia River Pacific lamprey population in the Umatilla River (described in Close et al. 2009).

**Table 1:** Summary information on Pacific lamprey sampling at Bonneville Dam

	2008						2009					2010				
			Genetic						Genetic					Genetic		
Statistical week	Bonn. count	Biosample (*N*)	Random	Rate (%)	Fate Supp.	Trans.	Bonn. count	Biosample (*N*)	Random	Rate (%)	Fate Supp.	Bonn. count	Biosample (*N*)	Random	Rate (%)	Fate Supp.
20	7	–	–	–	–	–	8	–	–	–	–	1	–	–	–	–
21	161	–	–	–	–	–	170	–	–	–	–	6	–	–	–	–
22	316	4	4	1.3	0	0	640	0	0	0.0	0	2	1	0	0.0	1
23	280	12	6	2.1	0	0	975	23	20	2.1	0	100	11	2	2.0	3
24	198	9	4	2.0	0	0	612	22	12	2.0	2	286	26	6	2.1	6
25	1401	71	28	2.0	2	0	520	28	10	1.9	2	405	17	8	2.0	1
26	1684	174	35(1)	2.1	5	0	487	30	10	2.1	2	389	9	8	2.1	0
27	1680	271	35(1)	2.1	10	0	721	49	14	1.9	3	328	6	6	1.8	0
28	1267	89	25	2.0	5	0	613	25	12	2.0	1	763	7	7	0.9	0
29	1044	165	24(3)	2.3	3	17	1048	39	21	2.0	0	722	23	14	1.9	1
30	1957	140	37	1.9	4	27	881	52	18	2.0	0	687	22	15(1)	2.2	0
31	866	130	17	2.0	4	26	812	7	7	0.9	0	436	11	10(1)	2.3	0
32	967	71	19	2.0	3	0	344	0	0	0.0	0	623	17	13(1)	2.1	0
33	709	62	14	2.0	2	0	254	7	5	2.0	0	439	12	9	2.1	0
34	937	56	18	1.9	0	0	208	11	4	1.9	1	371	8	6(1)	1.6	1
35	345	56	7	2.0	0	0	109	2	2	1.8	0	130	3	3(2)	2.3	0
36	277	–	–	–	–	–	105	–	–	–	–	193	–	–	–	–
37	322	–	–	–	–	–	47	–	–	–	–	177	–	–	–	–
38	47	–	–	–	–	–	48	–	–	–	–	69	–	–	–	–
39	29	–	–	–	–	–	20	–	–	–	–	48	–	–	–	–
40	47	–	–	–	–	–	13	–	–	–	–	39	–	–	–	–
41	17	–	–	–	–	–	3	–	–	–	–	14	–	–	–	–
Total	14558	1310	273	2.0	38	70	8638	295	135	1.6	11	6228	173	107	1.8	13

The tallies of Pacific lamprey ‘Bonn. Count’ per statistical week (week 20 begins by May 9 and week 41 ends by October 12) are provided by the Fish Passage Center (http://www.fpc.org) as observed by the Corps of Engineers at the Bonneville Dam fish counting window. For each year, we indicate the total number of fish that were tagged (Biosample *N*) and the subset of fish that were tissue sampled (Genetic) and used for a random sample, for supplementing spatial fate categories ‘Fate supp.’, and for a translocation ‘Trans.’. The weekly sample ‘Rate’ was calculated for the random sample. The weekly numbers for the random sample also indicate in parentheses the number of fish that did not have fate information.

**Table 2:** Sample sizes per spatial category of Pacific lamprey captured at Bonneville Dam and tracked via radiotelemetry

	Collection (*N*)			Genetic analysis (*N*)			
Final detection reach	2008	2009	2010	2008	2009	2010	Total
Below Bonneville	780	208	100	197	92	60	349
Bonneville to John Day (COL)	253	66	38	47	32	27	106
Bonneville to John Day (trib)	20	12	18	20 (19)	12 (6)	18 (10)	50 (35)
Above John Day (COL)	120	10	9	34 (12)	9 (5)	8 (3)	51 (20)
Above John Day (SNR)	8	1	1	8 (7)	1	1	10 (7)
Total	1181	297	166	306 (38)	146 (11)	114 (13)	566 (62)

A total of 62 fish were used to supplement a random sample to obtain a minimum of 50 fish in each major fate category. The number of supplemented fish included in each of the cell subtotals are indicated in parenthesis. The final detection category of Bonneville to John Day was further divided into fish that were last detected in the mainstem Columbia River (COL) or secondary tributary (trib). The Above John Day category could be further divided by those fish that reached the Snake River (SNR, above Ice Harbor Dam) versus those last detected in the Columbia River (COL).

As a first step to identify loci having significant associations with three main categories of predictor variables (migration timing, morphology, and migration fate), we performed univariate analyses using a general linearized model (GLM) and a mixed linearized model (MLM) with TASSEL v. 5.0.8 (Bradbury et al. 2007). The GLM is a fixed effects linear model that is utilized in TASSEL to identify significant associations between phenotypes and genotypes. TASSEL takes population structure into account by using membership in underlying populations as covariates in the model. The MLM is similar to GLM but includes both fixed and random effects (i.e., relationships among individuals) and can account for both population structure and kinship to improve statistical power (Yu et al. 2006). Principle components of the 85 neutral SNPs (first three PC axes) and a kinship matrix (‘scaled IBS’ method; Endelman and Jannink 2012) based on all 94 SNPs were generated in TASSEL to represent population structure and cryptic familial relationships, respectively. The MLM was implemented using the ‘P3D’ (Zhang et al. 2010) parameter option to shorten computation time, and the ‘without compression’ option to retain full dimensionality of the kinship matrix (each individual belongs to its own separate group). This latter option for the MLM provides results that are in extreme contrast to the GLM results, which effectively represent a ‘maximum compression’ option because it treats all individuals as a single group. Permutation tests (1000) were used to calculate *P*-values to determine significant associations of SNPs with traits. Due to the high power of this test to identify associations when many SNPs, traits, and individuals are included, a Bonferroni correction was applied to alpha levels (0.05, 0.01, and 0.001; e.g., 8.87 × 10^−5^ = corrected alpha 0.05) to reduce false positives and stringently control for Type I errors. The association tests using a GLM to test 6 traits were performed on the following two types of data samples: (i) total ‘Bonneville’ sample using PC axes and year as covariates, and (ii) the ‘Bonneville’ sample separated into 3-year samples that were each analyzed using PC axes as covariates. These two types of data samples were also analyzed using an MLM, and a kinship matrix was included as an additional covariate.

There were six traits included in all the association tests performed with TASSEL. Missing trait data (0–4.5% of data) were imputed by default parameter settings which uses an average of three nearest neighbor values. Migration timing was tested in units of the statistical week in which Pacific lamprey were collected and sampled at Bonneville Dam. Morphology predictor variables included Distell Fatmeter readings, girth, length, and weight. For the purposes of association testing, the river reach where a fish was last detected and its associated distance units were used to represent migration fate. Migration fate consisted of four river reaches: downstream from Bonneville Dam (235.1 km), between Bonneville Dam and John Day Dam (291.0 km), above John Day Dam (346.9 km), or above Ice Harbor Dam (i.e., near mouth of the Snake River, 537.7 km). In all analyses aside from association testing, fish that were last detected above Ice Harbor Dam were pooled with those detected above John Day Dam to increase sample size of this extreme upstream migration fate category.

A multivariate analysis (DISTLM*forward*, McArdle and Anderson 2001) implemented using the program package PRIMER version 6 and the PERMANOVA+ add-on (http://www.primer-e.com; Plymouth Marine Laboratory, Plymouth, UK) was used to examine the relative contribution of each predictor variable to explain variation at the SNP loci identified by the GLM and MLM association tests. Intercorrelation among these variables was examined (based on Pearson's *r*) to avoid excessive redundancy of predictor variables (|*r*| > 0.95), and *P*-values were calculated (SAS Institute, Inc. 2000). This multivariate approach was applied to the total ‘Bonneville’ sample. DISTLM*forward* was used for modeling the relationship between a resemblance matrix (i.e., a Euclidean distance matrix of genotypes for a particular SNP locus) and multiple predictor variables. Genotypes at SNP loci were converted to 0, 1, and 2 based on the number of minor alleles present, and the distance matrix was generated from the absolute value of the difference between all pairwise comparisons of individuals. The forward selection procedure fits individual environmental predictor variables or sets of predictor variables sequentially in the linear model. In our case, we used the following covariates and predictor variables which were the same as in the previous GLM tests: the first three principle components of the 85 neutral SNPs (‘PC’), year, fate, statistical week of passage (‘week’), Fatmeter (‘fat’), girth, length, and weight. We used 9999 permutations of the residuals under a reduced model to test the null hypothesis of no relationship (Anderson 2003). First, marginal tests were conducted on each predictor variable individually. Next, conditional tests were then performed using a stepwise forward selection procedure that identifies the most informative predictor variables sequentially while holding constant the variables already selected. Similar to the GLM association tests, we treated PC and year as covariates by forcing their inclusion into the model to focus on the other six predictor variables of interest (fate, week, fat, girth, length, and weight) which were added sequentially. The model ‘Akaike's Information Criterion’ (AIC, Akaike 1973) was used as the selection criterion in the sequential tests because it imposes a penalty for increases in each predictor variable, resulting in the most parsimonious model.

After demonstrating the importance of length and fate predictor variables, we categorized the Pacific lamprey with complete fate and length data (*N* = 566) into 12 collections, each fate category was divided into a short- and long-bodied collection (above and below the median size 660 mm, Fig. S1), and the ‘Below Bonneville Dam’ category was further split by year due to the ample number of samples represented by that fate category. The ‘Between Bonneville Dam and John Day Dam’ category could also be further split based on whether fish were last detected in a secondary tributary within this reach or not. In addition, we categorized 70 translocation Pacific lamprey into three collections (by statistical week), and included all newly categorized collections in a factorial correspondence analysis (GENETIX v. 4.03, Belkhir et al. 2004) to examine the relationships among collections.

We estimated proportions of particular categories of genotype, length class, and upstream fate to characterize a time series (time units in statistical weeks) of randomly sampled Pacific lamprey pooled across the 3 years (2008–2010). These estimated proportions allowed us to portray how seasonal differences in these proportions could potentially affect the composition of Pacific lamprey used for translocations. Specifically, the lengths and genotypes (based on pairwise *F*_ST_) of translocated fish that were taken during a time period that spanned three statistical weeks (29–31) in the year 2008, were compared with the following groups of fish: (i) Pacific lamprey that were randomly sampled during the same three statistical weeks across 3 years, (ii) Pacific lamprey that were randomly sampled during an earlier set of statistical weeks (25–27) across 3 years, and (iii) Pacific lamprey that were categorized into three major upstream fate categories. Significance testing of pairwise *F*_ST_ values was corrected for multiple testing using the B–Y method FDR as modified by Narum (2006) as this method controls Type I errors yet provides improved power to differentiate populations over Bonferroni correction.

Finally, we performed analyses on the 150 Pacific lamprey collected at Willamette Falls (Hess et al. 2014a) that had previously been tested for phenotypic associations of the adaptive loci. These fish only had length and passage-timing (units by statistical ‘week’) information, and we pooled these data with the total ‘Bonneville’ sample to perform association testing on this broader scale between the two regions (sample referred to as ‘Bonn + Willamette’). Two predictor variables were used in TASSEL (length and week) to identify loci with significant associations. Both GLM and MLM were performed, which required using TASSEL to first generate principle coordinates (first three PC axes based on the 85 neutral SNPs) to represent population structure and a kinship matrix (based on all 94 SNPs) to represent familial relationships. The GLM was performed using the following three covariates: PCs, location (0 = Willamette, 1 = Bonneville), and year. For the MLM, we used the same covariates as in the GLM, and additionally used the kinship matrix as a covariate. We further examined the significant loci using marginal tests in PRIMER (five covariates and predictor variables: PCs, location, year, length, and week). Finally, we used PRIMER to conduct multivariate analyses. Similar to the GLM in TASSEL, we treated the PCs, location, and year as covariates by forcing their inclusion in the multivariate model, and we implemented forward sequential tests in PRIMER to determine the model with the best fit using the remaining two predictor variables (length and week).

## Results

### HWE deviations

Linkage disequilibrium was statistically significant (adjusted alpha of 0.05 = 1.77 × 10^−4^) in three cases involving the following two pairs of loci: *Etr_384* × *Etr_1806* in Pacific lamprey from the random 2008 and 2009 sample, and *Etr_1806* × *Etr_4281* in the random 2009 sample. These three cases involved only adaptive loci that had not been found to be linked in previous analyses across a broader geographic range (Hess et al. 2013, 2014a). Further, we retained all loci for this study, because our downstream analyses found differences in their degrees of association with predictor variables.

Only one SNP locus (*Etr*_673) deviated from HWE (adjusted alpha of 0.05 = 1.77 × 10^−4^) in terms of large positive *F*_IS_ values, which were significant in both 2008 and 2009 random samples (*F*_IS_ = 0.289 and 0.353, respectively). This locus was retained for our analyses as HWE deviations can be an indication of selection; however, none of our downstream analyses indicated statistically significant associations of this locus with the predictor variables. Further, none of our previous analyses (Hess et al. 2013, 2014a) have indicated similar deviations in *F*_IS_ for other parts of the Pacific lamprey range.

### Association testing

The univariate GLM analyses performed in TASSEL identified three SNP loci (Etr_1806, Etr_4281, and Etr_5317) with significant associations (i.e., < Bonferonni adjusted alpha level of 0.001) with the girth, length, and weight predictor variables (‘Bonneville’ sample, Table 3). The MLM results were similar to the GLM but appeared to be more conservative, because two of the same SNPs (Etr_1806 and Etr_5317) were significantly associated with the same three predictor variables (< Bonferonni adjusted alpha level of 0.05), but Etr_4281 was no longer significantly associated with any traits. This difference likely owes to the extra covariate (kinship matrix) that results in the MLM test avoiding more false positives, at the expense of increasing false negatives.

**Table 3:** Test results using GLM and MLM in TASSEL which show the significant associations between SNP loci and six predictor variables

			Predictor variables					
Covariates	Sample	Locus	Fate	Week	Fat	Girth	Length	Weight
Year + PC (+ kinship)	Bonneville	Etr_1806				*** (*)	*** (**)	*** (*)
		Etr_4281					***	***
		Etr_5317				*** (***)	*** (***)	*** (***)
PC (+ kinship)	2008	Etr_1806				***	*** (**)	*** (*)
		Etr_4281					*	
		Etr_5317				*** (***)	*** (***)	*** (***)
PC (+ kinship)	2009	Etr_1806					**	
		Etr_4281					***	*
		Etr_5317					***	*
PC (+ kinship)	2010	Etr_1806						
		Etr_4281						
		Etr_5317				*** (**)	**	*** (*)
Year +	Bonn +	Etr_1806	NA		NA	NA	*** (*)	NA
Location	Willamette	Etr_2334	NA		NA	NA	**	NA
+ PC (+ kinship)		Etr_4281	NA		NA	NA	***	NA
		Etr_5317	NA		NA	NA	*** (***)	NA

Bonferroni correction was applied to alpha levels (0.05 ‘*’, 0.01 ‘**’, and 0.001 ‘***’), and were used to provide statistical robustness in identifying significant *P*-values for associations between traits and loci. *P*-values are shown for results from the GLM tests, and *P*-values from MLM tests are in parentheses. Covariates utilized in GLM tests are indicated and the same covariates were used in MLM with the addition of a kinship matrix (+ kinship). Covariates included principle coordinates (PC) of variation at 85 neutral SNPs as a proxy for population structure. The analyses were performed on the following three types of samples: the total ‘Bonneville’ sample, the ‘Bonneville’ sample split into 3 year samples using all six trait predictor variables, and performed on the ‘Bonn + Willamette’ sample excluding four variables with incomplete information (NA).

The TASSEL GLM results from the ‘Bonneville’ sample that was split into three separate year samples showed there were some interannual differences in the number of significant associations between 3 SNPs (Etr_1806, Etr_4281, and Etr_5317) and three morphological traits (girth, length, and weight; Table 3). For two of the year samples (2008 and 2009), all three SNPs were significantly associated with at least one morphological trait (length, < Bonferonni adjusted alpha level of 0.05); however, in 2010, only Etr_5317 was found to be significantly associated with morphological traits. The more conservative MLM results, demonstrated only 2 SNPs (Etr_1806 and Etr_5317) to be significant in 2008 and 1 SNP (Etr_5317) in 2010; in these cases, all three morphological traits (girth, length, and weight) were involved. The interannual differences (i.e., number of significant associations) likely owe to the differences in sample sizes which were lower with each subsequent year. In general, the morphological trait associations were most robust across year samples and across GLM and MLM tests for 2 SNPs (Etr_1806 and Etr_5317).

The TASSEL GLM analysis of the ‘Bonn + Willamette’ sample also identified *Etr_1806*, *Etr_4281*, and *Etr_5317* as loci with significant association with one of the morphological predictor variables (< Bonferroni corrected alpha 0.001 for length, Table 3). Further, there was a fourth locus, Etr_2334, identified to be significantly associated with length (< Bonferroni corrected alpha 0.01). Similar to previous analyses with the ‘Bonneville’ sample, only 2 loci (Etr_1806 and Etr_5317) were significant in the MLM analyses. Examination of intercorrelation of the six total predictor variables indicated that many of the morphological predictor variables were moderately to highly correlated with each other; however, none of them had a significant correlation that was above 0.95 (Table 4), and so all of the variables were retained for the multivariate analyses.

**Table 4:** Inter-correlation of predictor variables, Pearson's *r*

	Week	Fate	Fat	Girth	Length	Weight
Week				***	***	***
Fate	−0.0552			***	***	***
Fat	−0.0556	0.0381		***	***	***
Girth	−0.2490	0.1626	0.1993		***	***
Length	−0.1945	0.2100	0.1508	0.7351		***
Weight	−0.2506	0.1748	0.2022	0.9029	0.8831	

Pearson's *r* values and *P*-values are on bottom and top triangles, respectively. All *P*-values <0.05 were also <0.001 alpha level (***).

The marginal tests for the ‘Bonneville’ sample that were performed as part of the multivariate analysis were largely consistent with the TASSEL GLM and MLM results. Morphological predictor variables girth, length, and weight were highly significantly associated (*P* < 0.001) with the three loci identified by TASSEL (Table 5). Length was consistently the predictor variable that explained the largest portion of genetic variation for each locus. However, there was one nonmorphological predictor variable that met the most stringent alpha threshold of 0.001 (fate), which was observed to have significant association with *Etr_1806*. Further, this association with the fate predictor variable represented the highest percent of variation (2.6%) explained by any predictor variable aside from the three main morphological variables, girth, length, and weight. For the ‘Bonn + Willamette’ sample, four predictor variables were highly significantly (*P* < 0.001) correlated with three loci (*Etr_1806*, *Etr_5317*, and *Etr_4281*). For each of these three loci, the four predictor variables (length, location, year, and week) were consistently ranked from highest to lowest, respectively, based on percent variation explained (Table 5). The locus Etr_2334, in contrast, was significantly correlated with a single predictor variable (length, *P* < 0.01).

**Table 5:** Results from marginal tests of predictor variables and genetic variation of adaptive SNPs

		Locus							
		Etr_1806		Etr_2334		Etr_4281		Etr_5317	
Sample	Variable	%Var	*P*	%Var	*P*	%Var	*P*	%Var	*P*
Bonneville	PC	0.1		NA	NA	0.2		0.9	
	Year	0.1		NA	NA	0.0		0.4	
	Week	1.6	**	NA	NA	0.0		0.3	
	Fate	2.6	***	NA	NA	0.6		1.2	**
	Fat	0.3		NA	NA	0.3		0.3	
	Girth	6.8	***	NA	NA	2.2	***	15.8	***
	Length	12.2	***	NA	NA	7.2	***	17.0	***
	Weight	9.1	***	NA	NA	4.1	***	16.7	***
Bonn + Willamette	Location	9.8	***	0.0		10.1	***	34.2	***
	Year	7.0	***	0.0		7.8	***	24.8	***
	PC	0.1		0.7		0.1		0.6	
	Week	2.8	***	0.0		3.0	***	6.8	***
	Length	18.8	***	1.3	**	14.6	***	35.5	***

For each locus, the percent variation (%Var) explained by a particular predictor variable is provided. The (*P*) values from the marginal test were calculated from 9999 permutations, and indicated if below the following alpha levels: 0.05 (*), 0.01 (**), and 0.001 (***). The Bonn + Willamette sample combined data from two locations (Bonneville Dam and Willamette Falls) and fewer predictor variables for morphology were available to test, as compared to the ‘Bonneville’ sample. Only SNP loci that were identified with significant associations in the GLM and MLM tests in TASSEL (Table 3) were included in marginal tests for each sample, hence the missing data ‘NA’ for Etr_2334 in the ‘Bonneville’ sample.

The main pattern observed from the multivariate sequential tests on the ‘Bonneville’ sample showed length was the primary predictor variable that explained the largest percent of variation in the loci (range of 7–17%), followed by a combination of secondary predictor variables that were selected for the best fitting multivariate model, although these secondary variables explained <2% of the residual variation (Table 6). For example, weight and girth were selected as secondary predictor variables for *Etr_4281* and *Etr_5317*, respectively; fate and week were secondary predictor variables chosen for the best fitting multivariate model for *Etr_1806*. Finally, the analysis of the ‘Bonn + Willamette’ sample, which was conditioned on location, year, and PC axes, identified length as the only additional predictor variable required for the best fitting model in sequential tests (Table 6).

**Table 6:** Results from sequential tests of predictor variables and genetic variation at adaptive loci

Sample	Locus	Variable	AIC	SS (trace)	Pseudo-F	*P*	Prop.	Cumul.	Res. df
Bonneville	Etr_1806	Length	−980.9	14.2	78.8	***	0.121	0.123	570
		Fate	−984.4	1.0	5.4	*	0.008	0.131	569
		Week	−984.6	0.4	2.2	0.1358	0.003	0.134	568
	Etr_4281	Length	−706.6	12.9	44.6	***	0.072	0.075	570
		Weight	−707.8	0.9	3.2	0.0742	0.005	0.080	569
	Etr_5317	Length	−1200.4	14.1	114.1	***	0.165	0.177	570
		Girth	−1211.8	1.6	13.4	***	0.019	0.196	569
Bonn + Willamette	Etr_1806	Length	−1045.6	19.5	83.1	***	0.093	0.193	720
	Etr_2334	Length	−755.6	6.5	18.4	***	0.025	0.034	720
	Etr_4281	Length	−828.5	15.4	48.6	***	0.057	0.159	720
	Etr_5317	Length	−1261.1	20.3	115.9	***	0.091	0.437	720

Predictor variables are listed in the order they were selected to for a best fitting, forward sequential multivariate model using AIC as the selection criterion with the software PERMANOVA. Sum of squares (SS), the Pseudo *F*-value (Pseudo-F), the permutational *P*-value (*P*), proportion of variation (Prop.) and cumulated variation (Cumul.), and degrees of freedom (df) are shown. The Bonn + Willamette sample combined data from two locations (Bonneville Dam and Willamette Falls), and these sequential tests were conditioned on population structure (principle coordinates ‘PC’), year, and location to test the two remaining predictor variables, length and statweek. There were a total of six predictor variables analyzed in sequential testing with the ‘Bonneville’ sample, and sequential tests were conditioned on PC and year covariates (Table 5). The *P* values were calculated from 9999 permutations, and indicated if below the following alpha levels: 0.05 (*), 0.01 (**), and 0.001 (***).

### Morphology and fate

**Figure 2:**
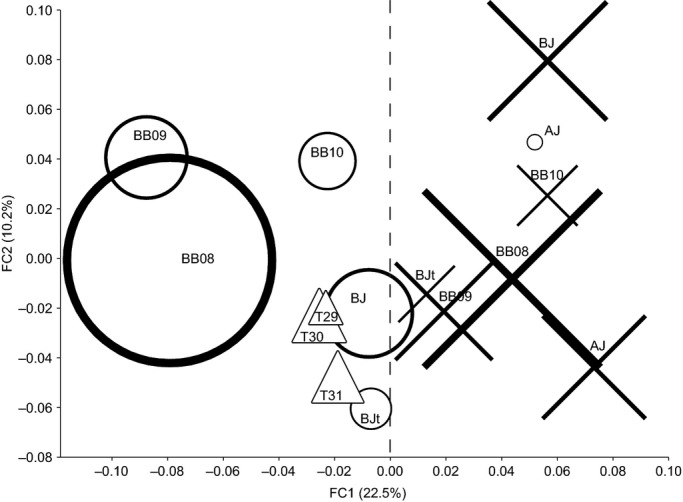
Factorial Correspondence Analysis of genotyped Pacific lamprey collections of short-bodied (<660 mm, circles) and long-bodied (>660 mm, X's) individuals from three upstream locations in the Columbia River. Individuals that traveled no further than below Bonneville Dam (BB) were further split into 3 years in which they were collected 2008–2010. Individuals that traveled upstream between Bonneville Dam and John Day Dam and were last recorded in the main stem Columbia River (BJ or in secondary tributaries BJt) and those that traveled above John Day Dam (AJ) were pooled together from three migration years. The AJ fate in this case included 10 fish that were detected further upstream above Ice Harbor Dam. Three Pacific lamprey collections from 2008 that were used in translocations (T, triangles) to the Umatilla River (above John Day Dam) were separated by statistical week (29, 30, and 31; Table 1). The dashed line is shown to emphasize the genetic differences between short-bodied and long-bodied collections.

Based on the results from association testing, we determined that length was the primary predictor variable that could explain genetic differences among individuals. To demonstrate this relationship, we examined a factorial correspondence analysis (FCA, Fig. 2) of our ‘Bonneville’ sample of Pacific lamprey that was recategorized into groups according to their length class (above and below the median of 660 mm, Fig. S1) and fate. This FCA plot clearly shows that groups of the long- versus short-bodied individuals segregate to different halves of the plot, regardless of their upstream fate category. The exception was the group of short-bodied Pacific lamprey from the above John Day Dam fate (the most upstream fate category), which may have been influenced by small sample size (*N* = 8). The group of translocated fish were also included in this FCA plot and they clustered closer to the short-bodied collections, despite the fact that these fish were ultimately released in a tributary upstream from John Day Dam.

### Seasonal heterogeneity of morphological and genetic traits in Pacific lamprey

**Figure 3:**
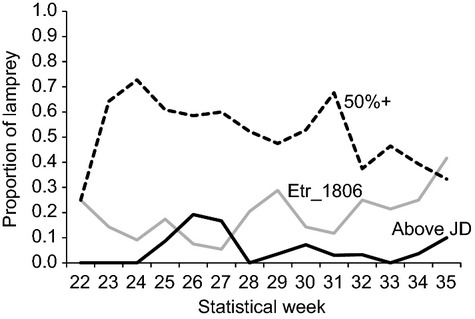
Weekly proportions of Pacific lamprey for presence of minor allele at Etr_1806 (gray), >50th percentile of length (dashed), and final upstream fate above John Day Dam (black), across a 3-year period 2008–2010. The above John Day Dam fate in this case included fish that were detected further upstream above Ice Harbor Dam.

The proportion of long-bodied fish generally decreased by statistical week throughout the season, but had a peak around week 31 (Fig. 3). Further, the weekly proportions of the minor allele at the locus *Etr_1806* (used to exemplify the adaptive loci) appears to contrast the pattern observed in the weekly proportions of Pacific lamprey with the most upstream fate category (above John Day Dam/Ice Harbor Dam), appearing to peak when the other declines and vice versa. The heterogeneous nature of Pacific lamprey throughout the migration season may explain some of the notable differences observed among various samples of fish that we compared. For example, based on morphological (length variation) and genetic (pairwise *F*_ST_ using only the three candidate SNPs from the ‘Bonneville’ sample analyses) comparisons, we observed some significant differences between the sample of translocated fish, and the two samples of randomly selected fish from different time periods in the migration season. The sample of translocated fish was significantly shorter than the sample of randomly selected fish from earlier in the season (statistical weeks 25–27), but showed a nonsignificant difference in length compared to the sample of randomly selected fish collected from the same time period (statistical weeks 29–31, Table 7). Average Pacific lamprey length was observed to be proportional with increasing distance traveled upstream, and the relatively short-bodied fish used in translocations were significantly shorter than all three samples of upstream fated fish. In terms of genetic composition, the translocated fish were not significantly different from either sample of randomly-selected fish, but *F*_ST_ was near 0.0 for the samples from the same time period, and near 0.01 for the samples from different time periods. Both the sample of translocated and later-season period randomly-selected fish were significantly genetically differentiated from the most upstream fate category (above John Day Dam/Ice Harbor Dam), which was not the case for the earlier-season period randomly selected fish.

**Table 7:** Influence of seasonality and final radio-tag detection location on the length and genetic traits of Pacific lamprey collections relative to Pacific lamprey used in translocations

				anova*P*-value			*F*_ST_		
Sample	Final reach	Statistical week	Average length	Translocation (29–31)	Random (29–31)	Random (25–27)	Translocation (29–31)	Random (29–31)	Random (25–27)
Translocation	–	29–31	648.6	–	0.224	**	–	−0.001	0.008
Random	–	29–31	656.1		–	**		–	0.010*
Random	–	25–27	668.6			–			–
Fate	BJ	–	663.7	**	0.084	0.465	0.009	0.008	−0.002
Fate	BJt	–	669.7	*	0.197	0.559	0.001	0.001	0.000
Fate	AJ	–	683.4	***	***	***	0.048*	0.043*	0.012

Lengths and genotypes of fish from the ‘translocation’ sample that were collected at Bonneville Dam during a time period that spanned three statistical weeks (29–31) in the year 2008 were compared (using anovas and *F*_ST_ for lengths and genotypes, respectively) to the following groups of fish from the total ‘Bonneville’ sample: (i) Pacific lamprey that were randomly sampled during the same three statistical weeks across 3 years, (ii) Pacific lamprey that were randomly sampled during an earlier set of statistical weeks (25–27) across 3 years, and (iii) Pacific lamprey that were spatially categorized by one of the following three final detection fates: between Bonneville Dam to John Day Dam in the main stem (BJ) and secondary tributary (BJt) and above John Day Dam (AJ). The AJ fate in this case included 10 fish that were detected further upstream above Ice Harbor Dam. *F*_ST_ was based on the three markers (*Etr_1806*, *Etr_4281*, and *Etr_5317*) associated with body morphology. Significant *F*_ST_ values (*) are indicated at a 0.05 alpha level corrected for multiple comparisons using B–Y method FDR (corrected alpha = 0.02041, Narum 2006). The significant anova*P*-values are indicated at the 0.05 (*), 0.01 (**), 0.001 (***) alpha levels.

## Discussion

### Morphological associations with genetic markers

Results from the current study clarified the adaptive significance of several loci that had been previously identified as outlier SNPs in a genomewide association study (GWAS) of Pacific lamprey (Hess et al. 2013). Morphology, primarily total adult body length, was found to have the strongest associations with genetic variation at three SNPs (*Etr_1806*, *Etr_4281*, and *Etr_5317*). The initial study by Hess et al. (2013) made it possible to categorize 4000 + loci as either putatively neutral or adaptive and further characterized which adaptive loci were significantly associated with several predictor variables (geography, dwarf body form, and run-timing). However, the opportunistic nature of the sampling for that study necessitated the inclusion of collections with incomplete metadata or lack of individual trait data. This constraint and the inter-correlation among predictor variables precluded the ability to determine a primary mechanism driving selection of the adaptive markers. Subsequent study by Hess et al. (2014a) provided additional support for associations of two of these loci (*Etr_1806* and *Etr_5317*) with length and run-timing via analyses of samples with complete metadata on individuals; however, that study did not account for inter-correlation among predictor variables and interpretation was further complicated by the known presence of over-wintering behavior (Clemens et al. 2010; Hess et al. 2014a).

The current study was designed to resolve some of these issues and results demonstrated that the primary mechanism operating on three of these adaptive markers is related to adult body length of Pacific lamprey. Further, the primary association of body length with these markers appears to extend across a broad region that includes both Willamette Falls and Bonneville Dam, because association tests conditioned on location indicated that body length rather than the alternative predictor variable that was tested (run-timing) could explain a significant portion of the residual variation. However, while migration fate and timing play more secondary roles, they explain minor but significant portions of the remaining genetic variation based on the Bonneville Dam samples. These candidate loci may be associated with the genetic mechanisms underlying the size differences observed among migrating Pacific lamprey. Because body size is associated with the distance Pacific lamprey migrate upstream (Keefer et al. 2009, 2013a), this relationship appears to explain why Pacific lamprey found in the interior Columbia River basin are genetically differentiated at these loci from those that spawn in the lower basin. We note that while our metric of body size (length) was correlated with other size metrics (weight, girth), it seems plausible that other unmeasured factors related to both size and morphology (i.e., shape) also affect migration distance.

### Functions of genes associated with genetic markers

Although the genetic mechanisms associated with each of these three adaptive loci (*Etr_1806*, *Etr_4281*, and *Etr_5317*) are likely complex, we have some information regarding the reported functions of the genes to which the SNPs were localized. For example, *Etr_5317*, localizes to the gene DYM (Hess et al. 2013), which encodes a protein necessary for normal skeletal development and brain function (El Ghouzzi et al. 2003; Denais et al. 2011). The SNP locus, *Etr_4281*, aligns with the human homolog, PCDH15 (Hess et al. 2013), which is in the cadherin gene superfamily and encodes integral membrane proteins that mediate calcium-dependent cell–cell adhesion. Functions include an essential role in maintenance of normal retinal and cochlear function and mutations in this gene result in hearing loss in humans (Ahmed et al. 2008). The other SNP locus, *Etr_1806,* which was highly correlated with Pacific lamprey morphology, does not appear to localize within any described genes but occurs approximately 15 kb from a genomic region conserved between sea lamprey and lancelet (*Branchiostoma floridae,* Hess et al. 2013). Finally, the SNP locus, *Etr_2334*, appears to be a cryptic adaptive locus that had previously been categorized as putatively neutral (Hess et al. 2013), but results from the merged sample ‘Bonn + Willamette’ found significant association with length. Further, this locus was found to localize to a genomic region in sea lamprey that is homologous to the human gene, CC2D2A, which plays a critical role in cilia formation and development (Gorden et al. 2008; Doherty et al. 2010).

Insight into the genetic mechanisms associated with the three main candidate loci in this study may also be gained by examining homologous genes and functions associated with the groups of loci that have previously been found by Hess et al. (2013) to be highly linked to these 3 SNPs. For example, the SNP locus *Etr_5317* was part of a group of 53 linked loci (group A) that were identified in the GWAS (Hess et al. 2013). This locus was observed in significant linkage with 98% of loci in the lower Columbia, 96% in the interior Columbia, and with 75% and 94% outside the Columbia River to the north and south, respectively (Hess et al. 2013). Functions associated with genes in which these 53 SNPs loci localize include involvement in cartilage development (CILP, Nakamura et al. 1999; *Etr_5340*), and resistance to oxidative stress (SZT2, Basel-Vanagaite et al. 2013; *Etr_1489*). In addition, studies of gene defects (Kalay et al. 2011) suggest involvement in seckel syndrome type 5, a rare autosomal recessive disorder characterized by proportionate dwarfism (CEP152, *Etr_68*), and acromesomelic dysplasia (Robinson et al. 2013) which is an extremely rare, inherited, progressive skeletal disorder that results in a particular form of short stature (i.e., short-limb dwarfism, NPR2, *Etr_6363*).

The SNP locus *Etr_1806* was part of another group of 25 linked loci (group B), and this SNP had been observed in significant linkage with all 25 loci in the lower Columbia, and 67% of group-B loci in the interior Columbia but was linked to far fewer loci (0% and 13% in northern and southern regions, respectively) outside the Columbia River (Hess et al. 2013). Functions associated with genes in which these markers are located include lower limb spasticity (KIF1C, Dor et al. 2014; *Etr_4193*), axonemal motor/ATPase activity (DNAHC8, Samant et al. 2002; *Etr_393*), stabilization of dynamic microtubules (CLASP2, Maia et al. 2012; *Etr_1257*), muscle functioning (TRIM32, Frosk et al. 2002; *Etr_3295*), as well as relatively uncharacterized functions of a signaling protein (WAC, Xu and Arnaout 2002; *Etr_320*) and zinc finger protein (ZNF385D, Lamesch et al. 2007; *Etr_5213*).

Finally, the *Etr_4281* locus was identified as part of a group of 27 linked loci (group C). This locus was observed in significant linkage with 100% of the group-C loci in the lower Columbia, and 96% in the interior Columbia, with 54% outside the Columbia River to the north and south (Hess et al. 2013). Functions (and loss of functions) associated with genes in which these group-C-SNP loci localize include signal transduction [RAPGEF2 (Emery et al. 2013), *Etr_746*; PCNA (Webb et al. 1990), *Etr_4051*; WDR91 (Caldwell et al. 2005), *Etr_5841*], craniofacial, urogenital, and respiratory system abnormalities (FRAS1, Hoefele et al. 2013; *Etr_2776*), myofibril assembly (NRAP, Mohiddin et al. 2003; *Etr_2963*), regulation of cardiovascular functions and relaxation of smooth muscle tone (PRKG1, Guo et al. 2013; *Etr_3055*), limb girdle muscular distrophy (TRAPPC11, Bögershausen et al. 2013; *Etr_6149*), as well as the uncharacterized function of a member of a methyltransferase gene superfamily (THUMPD2, Zhang et al. 2001; *Etr_856*).

The homologous genes and functions involved in body size and muscle development are most obviously relevant to the phenotypic associations that we identified for all four candidate SNPs (*Etr_1806*, *Etr_2334*, *Etr_4281*, and *Etr_5317*) within the Columbia River. It remains unknown to what extent the morphological associations of these loci may apply to Pacific lamprey outside the Columbia River basin. We hypothesize that SNPs that localize more proximally to the target of selection in the genome will likely have to be identified to observe this pattern more broadly because of breakdown in these phenotypic associations that was noted previously by Hess et al. (2014a).

### Mechanism of selection on morphology

At this point, we cannot determine whether the driving force of selection on the candidate SNPs originates from anthropogenic factors (e.g., main stem hydropower dams, altered hydrograph), or rather from selection due to the energetic requirements of lengthy upstream migration or the differences in habitat present in interior versus streams closer to the Pacific Ocean. However, it has been suggested there was greater morphological diversity (small-bodied adults were present) in the interior Columbia River basin historically (Close et al. 2004). The main stem Columbia River dams are known to impose substantial impediments to Pacific lamprey migration, as there is high attrition at Bonneville Dam and at each of the dams upstream, and tagged adults successfully passing the dams are consistently larger than those that do not (e.g., Keefer et al. 2009). Fishways at lower Columbia and Snake River dams were not designed for adult Pacific lamprey passage (Moser et al. 2011), and many adults fail to pass fishways (e.g., Keefer et al. 2013a,b). The genetic associations observed here could thus be caused by long-distance migration itself, selection for traits during difficult passage of artificial fishways, or both. The relationship between passage ability at fishway barriers and Pacific lamprey traits (especially size) is currently under investigation. If distance is a surrogate for migration investment, then passing dams may reduce numbers upstream by either imposing high energetic costs or directly impeding passage of Pacific lamprey with small body size at barriers requiring high swimming power and/or endurance. Evaluation of the relationships between migration distance, morphological traits, life history, and genetic variation in large unimpounded drainages such as the Fraser River in British Columbia would be especially useful for separating the effects of distance and dams on the gradients observed here. Regardless, the observed patterns raise important questions about anthropogenic effects on the composition of interior Pacific lamprey populations.

It may initially seem paradoxical to observe adaptive divergence that is driven by body size and upstream distance traveled without also observing significant differentiation at neutral loci within the Columbia River basin. The lack of neutral genetic differentiation among major rivers (e.g., Goodman et al. 2008; Hess et al. 2013) may be driven by non-specificity in choice of highly mobile hosts during its ectoparasitic feeding mode which results in wide dispersion in ocean waters (similar to sea lamprey, Waldman et al. 2008) and could subsequently be reinforced by selection against long return migrations to natal streams, a lack of sensory capacities to navigate and orient to natal streams, or other selective forces. Nonetheless, the apparent paradox may be explained by nonphilopatric migration, continuous distribution, historically high effective population size of this anadromous fish, and on-going selection for larger body size during long or difficult migration. While Pacific lamprey appear to segregate according to body length and upstream distance, they have low probability of spawning in their natal stream, which would allow sufficient gene flow throughout the range to homogenize the neutral variation of the population. This hypothesis could be tested by surveying genetic variation within tributaries distributed throughout the Pacific lamprey range. If the hypothesis were true, we would predict a consistent pattern within each tributary of a gradient of adaptive variation correlated with upstream distance and contrasted by minimal neutral structure at the same scale.

It is possible we have ignored other key factors that could influence body size and drive the adaptive divergence observed in this species. No methods are available to reliably determine age in lamprey, and thus, we were unable to evaluate how maturity, growth rate, and adult body size were related to the observed genetic associations, though we expect that factors affecting maturity in lamprey are under strong selection in this semelparous species. Further, body size at maturity may be affected by the duration of time in freshwater or ocean environments, or specialization on particular resources. Individuals originating from upstream locales (i.e., less productive streams) may reproduce at older ages and return to freshwater at large adult sizes. Alternatively, large-bodied Pacific lamprey may return to upstream sites because of some olfactory cue, as such cues have been demonstrated to influence migration behavior in Pacific lamprey (Yun et al. 2011) and other lamprey species (e.g., *Petromyzon marinus*; attractants, Li et al. 2002; and repellents, Wagner et al. 2011). However, any alternative scenario would effectively produce the same result that involves nonphilopatric, large-bodied adults having heightened propensity for migrating to upstream locales. It would be ideal if future exploration of drivers of selection on Pacific lamprey could greatly broaden the types of phenotypes measured at various life stages to adequately treat these possible alternatives.

### Adaptive genetic context for translocation strategies and migratory behavior

This study has demonstrated a seasonal effect on genetic and morphological diversity, and further showed that the manner in which Pacific lamprey are selected for translocation can result in significantly differentiated genetic and phenotypic variation as compared to individuals that volitionally migrated to the point of release. If anthropogenic factors are the primary mechanism driving selection on these volitional migrants, translocations of fish may be one way to restore historical levels of genetic and morphological diversity in the interior Columbia River basin. Alternatively, if it is driven by selection on factors such as migration distance, we would predict that translocations would serve to increase genetic diversity upon which natural selection would continue to act within the interior river basin.

Adaptive genetic markers that associate with migration potential in a migratory species open the possibility for predicting how individuals may distribute themselves across their range prior to their migration. Importantly, selecting lamprey for translocation that exhibit a favorable migration trait does not guarantee recruitment success. This will likely depend on whether the traits associated with adult migration are also linked to traits that improve adult overwintering and spawning success in interior streams, as well as growth and survival in early life history and during juvenile outmigration. Since adult Pacific lamprey with the largest body lengths migrate the longest upstream distances and at least three genetic markers in our panel are significantly associated with length, this combination of genetic and morphological characteristics may allow future prediction of fate for individual fish. This information could be exploited even at the very early life stages of Pacific lamprey to forecast, for example, from out-migrating juveniles, how abundance of adult Pacific lamprey may be distributed throughout the Columbia River basin many years in the future.

## Data archiving

Data for this study are available at the Dryad Digital Repository: http://doi.org/10.5061/dryad.t0391.

## Supporting Information

**Figure S1.** Length distributions of the Bonneville Dam and Willamette Falls samples of Pacific lamprey.
