# Supplementary material for: Genes predict long distance migration and large body size in a migratory fish, Pacific lamprey
Source: Evol Appl. 2014 Sep 23;7(10):1192–208. doi: 10.1111/eva.12203 (PMC4275091; doi:10.1111/eva.12203)
Supplement: Supplementary file 1 — Figure S1. Length distributions of the Bonneville Dam and Willamette Falls samples of Pacific lamprey. [file eva0007-1192-sd1.pdf]

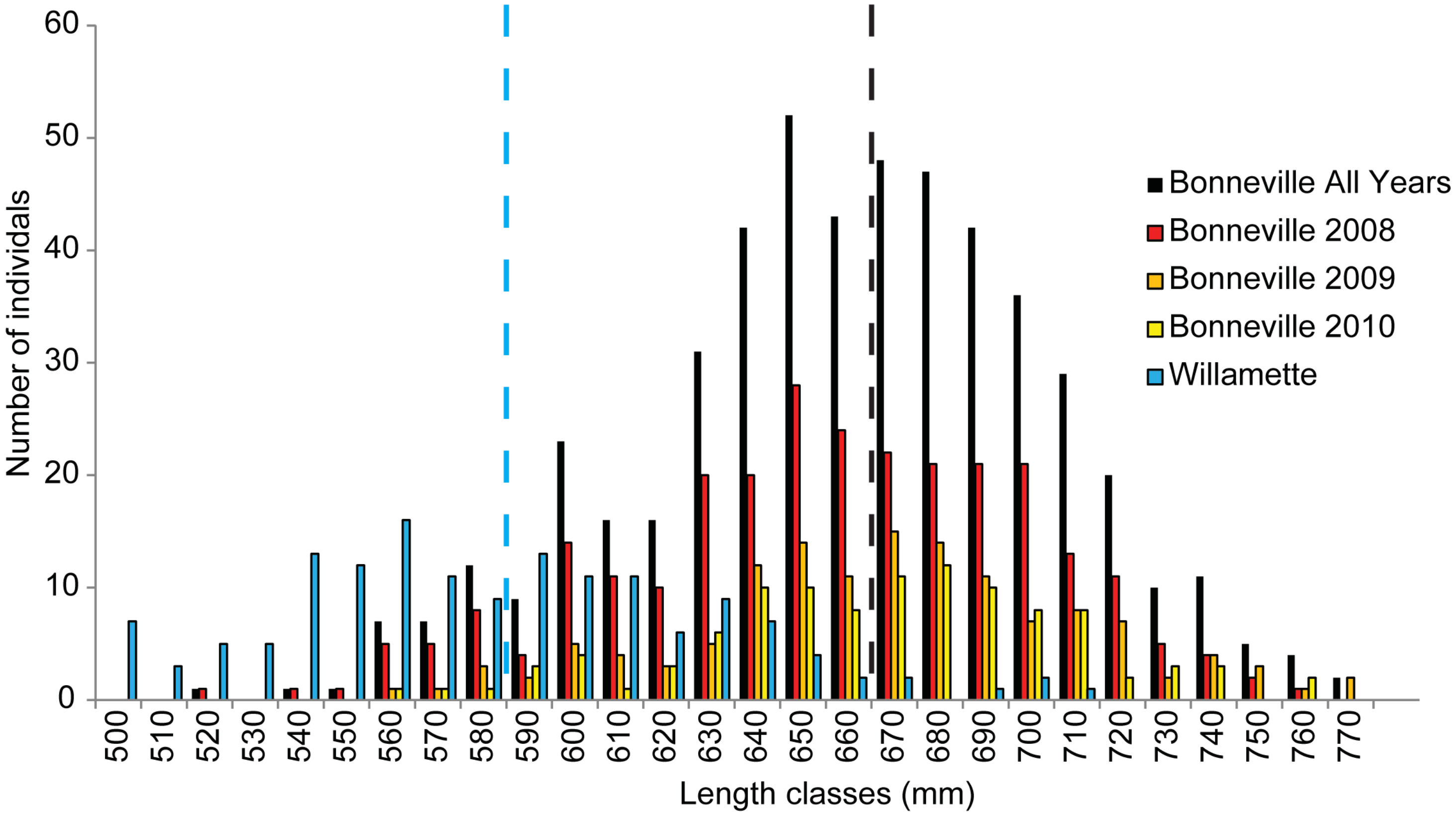

Figure S1. Length distributions of the Bonneville Dam and Willamette Falls samples of Pacific lamprey. The dashed lines indicate the median length of the Bonneville Dam all-years sample (660 mm, black line) and the Willamette Falls sample (575 mm, blue line).
